# Clinical manifestations of patients with *STAT1* gain-of-function variants in Mexico

**DOI:** 10.3389/fimmu.2026.1986964

**Published:** 2026-10-20

**Authors:** Tiareth Lisset Cova-Guzmán, Satoshi Okada, Miyuki Tsumura, Patricia María O´Farrill-Romanillos, Rubén Martínez-Barricarte, Carla Marina Román-Montes, Marco Antonio Yamazaki-Nakashimada, Luisa Berenise Gámez-González, Viridiana López-Martínez, Gina Maribel Meléndez-Díaz, Carlos Gerardo Zuñiga-Lagunes, Héctor Gómez-Tello, María Enriqueta Nuñez-Nuñez, Beatriz Bayardo Gutiérrez, Aidé Tamara Staines-Boone, Saúl Oswaldo Lugo Reyes, Yuridia Salazar-Gálvez, Cristina Moctezuma-Trejo, Angélica Kathya Alaníz-Flores, Noemi Gómez-Hernández, Margarita Ortega-Cisneros, María del Carmen Zárate-Hernández, Juan Carlos Bustamante-Ogando, Camille Soudée, Jérémie Rosain, Stéphanie Boisson-Dupuis, Mélanie Migaud, Jean Laurent Casanova, Francisco Javier Espinosa-Rosales, Giancarlo Hernán Cristerna Tarrasa, Deborah Palacios-Reyes, Anne Puel, Jacinta Bustamante, Sara Espinosa-Padilla, Lizbeth Blancas Galicia

**Affiliations:** 1 Laboratory of Immunodeficiency, National Institute of Pediatrics, Mexico City, Mexico; 2 Department of Pediatrics, Hiroshima University Hospital, Hiroshima, Japan; 3 Hiroshima University Graduate School of Biomedical and Health Sciences, Hiroshima, Japan; 4 Department of Allergy and Immunology, Siglo XXI National Medical Center, Mexican Social Security Institute (IMSS), Mexico City, Mexico; 5 Division of Genetic Medicine and Clinical Pharmacology, Department of Medicine, Vanderbilt Genetics Institute, Vanderbilt University Medical Center, Nashville, TN, United States; 6 Division of Molecular Pathogenesis, Department of Pathology, Microbiology, and Immunology, Vanderbilt Center for Immunobiology, Vanderbilt Institute for Infection, Immunology, and Inflammation, Vanderbilt University Medical Center, Nashville, TN, United States; 7 Clinical Microbiology Laboratory, National Institute of Medical Sciences and Nutrition Salvador Zubirán, Mexico City, Mexico; 8 Infectious Diseases Department, National Institute of Medical Sciences and Nutrition Salvador Zubirán, Mexico City, Mexico; 9 Department of Immunology, National Institute of Pediatrics, Mexico City, Mexico; 10 Department of Immunology, Hospital Infantil de Especialidades de Chihuahua, Chihuahua, Mexico; 11 Department of Internal Medicine, Hospital General de México, Mexico City, Mexico; 12 Department of Immunology and Allergy, Angeles Hospital, Tijuana, Mexico; 13 Department of Immunology, Hospital de la Niñez Poblana, Puebla, Mexico; 14 Department of Allergy and Clinical Immunology Department, Nuevo Hospital Civil de Guadalajara “Dr. Juan I.Menchaca, Guadalajara, Jalisco, Mexico; 15 Department of Immunology, Unidad Médica de Alta Especialidad (UMAE) No. 25, Mexican Social Security Institute (IMSS), Monterrey, Nuevo León, Mexico; 16 Department of Allergy and Clinical Immunology, UMAE “La Raza”, Mexican Social Security Institute (IMSS), Mexico City, Mexico; 17 Department of Immunology, Centro Médico de Occidente, Mexican Social Security Institute (IMSS), Jalisco, Mexico; 18 Regional Center of Allergy and Clinical Immunology, Hospital Universitario “Dr. José Eleuterio González”, Monterrey, Mexico; 19 Université Paris Cité, Imagine Institute, Laboratory of Human Genetics of Infectious Diseases, Necker Branch, Institut National de la Santé et de la Recherche Médicale U1163, Necker Hospital for Sick Children, Paris, France; 20 Laboratory of Human Genetics of Infectious Diseases, University of Texas Southwestern Medical Center, Dallas, TX, United States; 21 Center for the Genetics of Host Defense, University of Texas Southwestern Medical Center, Dallas, TX, United States; 22 Department of Microbiology, University of Texas Southwestern Medical Center, Dallas, TX, United States; 23 Howard Hughes Medical Institute, New York, NY, United States; 24 Center for Immunology, Allergy, and Pediatrics, Hospital Ángeles Lomas, Mexico City, Mexico; 25 Department of Infectious Diseases, National Institute of Pediatrics, Ciudad de Mexico, Mexico; 26 Department of Mycology, National Institute of Pediatrics, Mexico City, Mexico; 27 Center for the Study of Primary Immunodeficiencies, Necker Hospital for Sick Children, AP-HP, Paris, France

**Keywords:** BCG, gain-of-function, histoplasmosis, HLH, mycobacteria, STAT1, tuberculosis

## Abstract

**Background:**

Germline Signal Transducer and Activator of Transcription 1 (STAT1) gain-of-function (GOF) variants enhance cellular responses to IL-27 and type I, II, and III interferons, predisposing to fungal, bacterial, and viral infections and immune dysregulation.

**Methods:**

Mexican patients with pathogenic STAT1-GOF variants identified at referral centers were enrolled.

**Results:**

Thirty patients from 17 kindreds were included. Fifteen variants were identified, including the novel C324W variant, confirmed as GOF by functional studies. All patients developed mucocutaneous candidiasis. Among 25 BCG-vaccinated patients, 24% developed BCG disease. According to the Graham criteria, seven patients (23%) with tuberculosis were classified as confirmed (n=1), possible (n=3), and probable (n=3) cases. Three patients developed both BCG disease and tuberculosis. Autoinflammatory and autoimmune manifestations occurred in 50% and 40%, respectively. Two patients developed infection-triggered macrophage activation syndrome. Seven patients (23%) died.

**Conclusions:**

This cohort illustrates the broad clinical spectrum of STAT1 GOF disease, including infectious, inflammatory, autoimmune, and hematological manifestations. BCG disease and tuberculosis were observed in a subset of patients, highlighting the complexity of clinical presentation and the importance of comprehensive evaluation and follow-up.

## Introduction

Signal Transducer and Activator of Transcription 1 (STAT1) gain-of-function (GOF) disease is an inborn error of immunity (IEI) caused by monoallelic pathogenic variants in the *STAT1* gene that enhance and prolong STAT1-mediated signaling (1–4). The clinical phenotype is highly heterogeneous, encompassing infectious susceptibility, autoimmunity, immune dysregulation, vascular complications, and chronic inflammatory manifestations. Infections are predominantly caused by fungal, bacterial, and viral pathogens (5). Chronic mucocutaneous CMC, resulting from impaired IL-17-mediated immunity, is the hallmark infectious manifestation and typically involves the oral cavity, skin, nails, and esophageal mucosa (6, 7). Viral infections frequently involve herpes simplex virus, varicella-zoster virus, human papillomavirus, Epstein–Barr virus, and cytomegalovirus, whereas invasive fungal occur less commonly (7–9). Recurrent bacterial infections mainly affect the upper and lower respiratory tract, presenting as sinusitis, otitis media, bronchitis, and pneumonia, most commonly caused by *Streptococcus pneumoniae*, *Haemophilus influenzae*, and *Staphylococcus aureus* (8). Mycobacterial infections have emerged as an additional infectious manifestation of STAT1 GOF disease, although they are considerably less frequent than CMC and recurrent bacterial respiratory infections (6, 8). Reports of both tuberculosis and Bacillus Calmette–Guérin (BCG) disease have broadened the recognized infectious spectrum of STAT1 GOF disease beyond fungal and pyogenic bacterial infections (6, 8). The frequency of mycobacterial infections appears to vary among published cohorts (10), and regional epidemiological factors, including tuberculosis prevalence and BCG vaccination policies, may contribute to differences in the clinical phenotype beyond the underlying immune defect (8). We describe a Mexican cohort from a region endemic for fungal infections, with a high burden of tuberculosis and universal neonatal BCG vaccination.

## Materials and methods

### Patients

Between 2015 and 2026, Mexican patients harboring pathogenic GOF variants in *STAT1* were identified at multiple referral centers throughout Mexico and included in this study. Demographic, clinical, microbiological, immunological, genetic, mycobacterial treatment, and outcome data were retrospectively collected through a review of medical records. Data were entered into a Microsoft Excel database by the treating physicians and subsequently analyzed. Descriptive statistics were used to summarize the data. Categorical variables are presented as frequencies and percentages, whereas continuous variables are reported as medians and interquartile ranges (IQRs). The study protocol and informed consent form were approved by the Institutional Review Board (019/2011) and the corresponding institutional ethics committees before study initiation. Written informed consent was obtained from all participants or their parents or legal guardians before enrollment.

### Immunological evaluation

Immunological assessment included measurement of serum immunoglobulin levels (IgG, IgA, IgM, and IgE) and analysis of T-cell subsets (CD3+, CD4+, and CD8+). Results were interpreted according to age-adjusted reference ranges.

### Genetic studies

*STAT1* variants were identified by Sanger sequencing an IEI gene panel or whole-exome sequencing (WES). Genomic DNA (gDNA) was isolated from the whole blood samples using the GeneJET Genomic DNA Purification Kit (Thermo Fisher Scientific, Waltham, MA, USA). Whole-exome sequencing (WES) was performed on an Illumina NovaSeq 6000 platform. Briefly, library preparation and capture were performed utilizing the XGen Research Panel probes from IDT according to the manufacturer’s instructions. Paired-end sequencing (2 × 150 bp) was conducted, targeting an average of 20 million reads per sample (~50× coverage). Bioinformatic analysis was performed using Webseq. DNA samples from family members were used to perform familial segregation analysis by Sanger sequencing. Variant nomenclature and classification followed the guidelines of the American College of Medical Genetics and Genomics (ACMG) and the Association for Molecular Pathology (AMP) (11). Genetic testing was performed at Vanderbilt University Medical Center, the Laboratory of Human Genetics of Infectious Diseases, and the National Institute of Pediatrics.

### Functional assays

The p.C324W (p.Cys324Trp) variant, initially classified as a variant of uncertain significance (VUS), was functionally characterized using a Gamma-Activated Sequence (GAS) reporter assay to evaluate its effect on IFN-γ-mediated STAT1 signaling. U3C cells were cotransfected with plasmids encoding wild-type (WT) *STAT1*, the C324W variant, the Y701C variant (loss-of-function control), the R274Q GOF variant as control, or an empty vector (mock), together with GAS reporter plasmids (Cignal GAS Reporter Assay Kit, QIAGEN). Following transfection, cells were stimulated with IFN-γ at concentrations of 10 or 1000 IU/mL for 16 hours. Firefly luciferase activity was normalized to Renilla luciferase activity in each well. Three independent experiments were performed with three wells per condition. The three well values were averaged within each experiment, and results are presented as mean ± SEM of the experiment-level means (n = 3). Statistical significance was assessed using the Holm–Bonferroni method.

### Definitions

Adverse reactions to BCG vaccination were classified as follows: localized BCG-itis was defined as a local abscess or severe ulcer at the site of injection, and regional BCG-itis was defined as the involvement of regional ganglia in the axillary, supraclavicular, and cervical areas, including enlargement, suppuration, and/or fistula formation. Disseminated BCG-osis was defined as the presence of BCG at more than 1 remote site, a positive blood or bone marrow culture, or both (12).

Tuberculosis infection was diagnosed according to the criteria proposed by Graham et al. (13). Basically, confirmed tuberculosis infection was defined as the presence of at least one sign or symptom suggestive of tuberculosis, with microbiological confirmation in the form of a positive culture for *Mycobacterium tuberculosis*. Patients with at least one sign or symptom suggestive of tuberculosis but negative cultures were classified as having probable or possible infection. Patients were considered to have a probable infection if they had one sign or symptom suggestive of tuberculosis, a chest radiograph consistent with intrathoracic tuberculosis, and at least 1 of the following (1): positive clinical response to antituberculosis treatment (2), documented exposure to tuberculosis or close contact with a patient with tuberculosis, or (3) immunologic evidence of *M. tuberculosis* in the form of a positive tuberculin skin test result or tuberculosis IFN-γ release assay. Possible tuberculosis was defined as a sign or symptom suggestive of tuberculosis infection and a positive clinical response to antituberculosis treatment but with a chest radiograph that was not consistent with pulmonary tuberculosis or documented exposure to tuberculosis, close contact with a patient with tuberculosis, or immunologic evidence of *M. tuberculosis* in the tuberculin skin test or IFN-γ release assay.

## Results

### Genetic and immunological features

We studied 30 patients from 17 kindreds originating from Mexico, who were identified and treated at 13 referral centers across the country (**Figure 1**). Twelve patients had been previously reported in the literature (6, 7, 14–17). The geographic distribution showed a predominance of patients from the south-central region of the country, accounting for 63.3% of cases (*n* = 19). The northeast region represented 13.3% (*n* = 4), while the eastern and northwestern regions each contributed 10.0% (*n* = 3). The southwest region accounted for the remaining 3.3% (*n* = 1) (**Figure 2**). Of the 30 patients, 50% were women and the rest were men. The global age at onset of clinical manifestations was not included because adult patients were unable to clearly recall this information. The median age of them at the time of this description was 28 years (IQR [interquartile range] 10-36). Nineteen of the 30 cases (63.3%) belonged to six kindreds and eleven cases were sporadic, with a single affected individual identified in each family (**Table 1**). The father of P30 (family Q), with a history of CMC, declined genetic testing and was therefore not included in this description.

**Figure 1:**
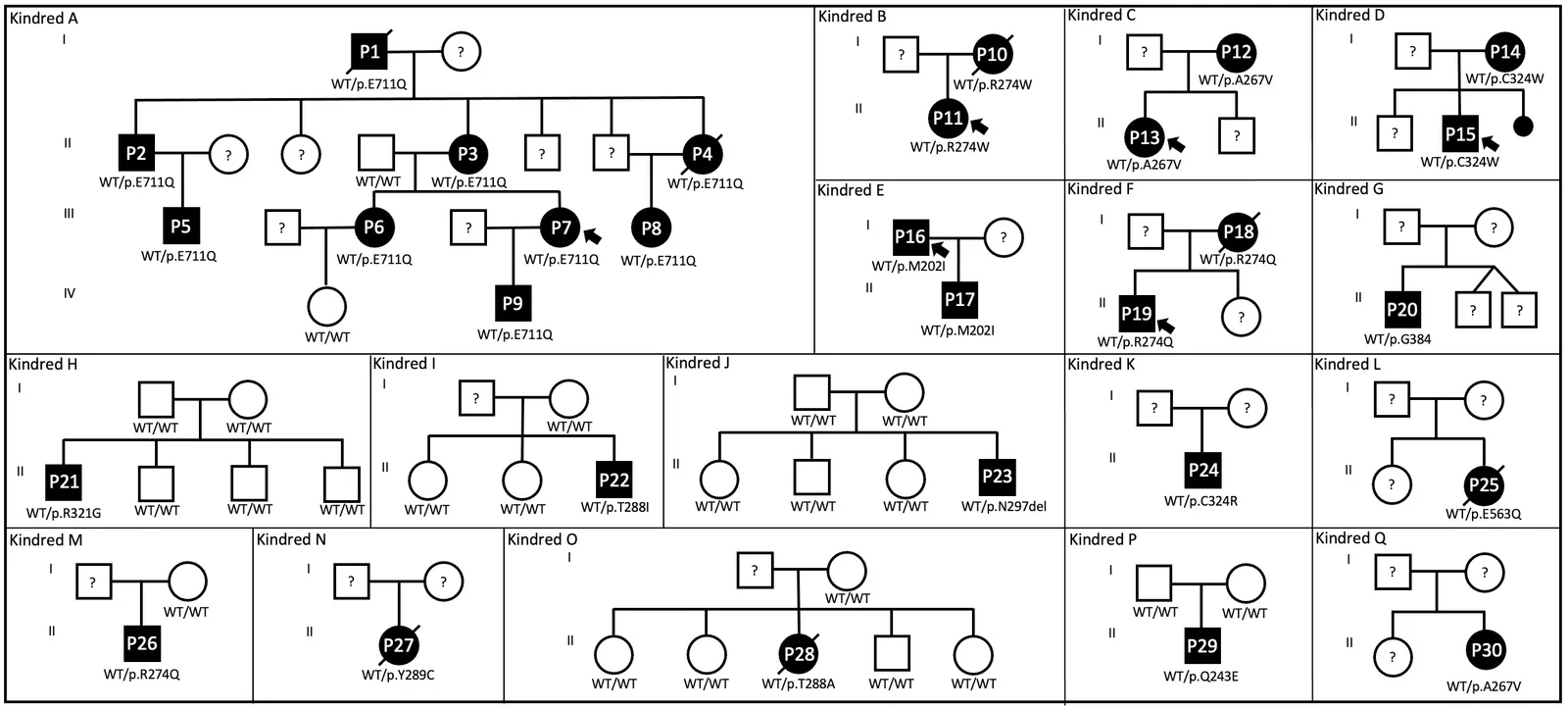
Pedigrees in 17 Mexican families with GOF STAT1 disease. Each family is identified by a letter **(A–Q)**. Generations are indicated by Roman numerals (I-IV). Individuals affected by *STAT1* GOF are shown in black. Probands are indicated by an arrow. Males are represented by squares and females by circles. The symbol “?” indicates individuals not genetically studied. WT wild-type allele. The pathogenic variant is indicated below each individual. A small, black-filled circle represents miscarriage. Figures with a diagonal line indicate deceased individuals. Twins are represented by two symbols joined by diagonal lines originating from the same point.

**Figure 2:**
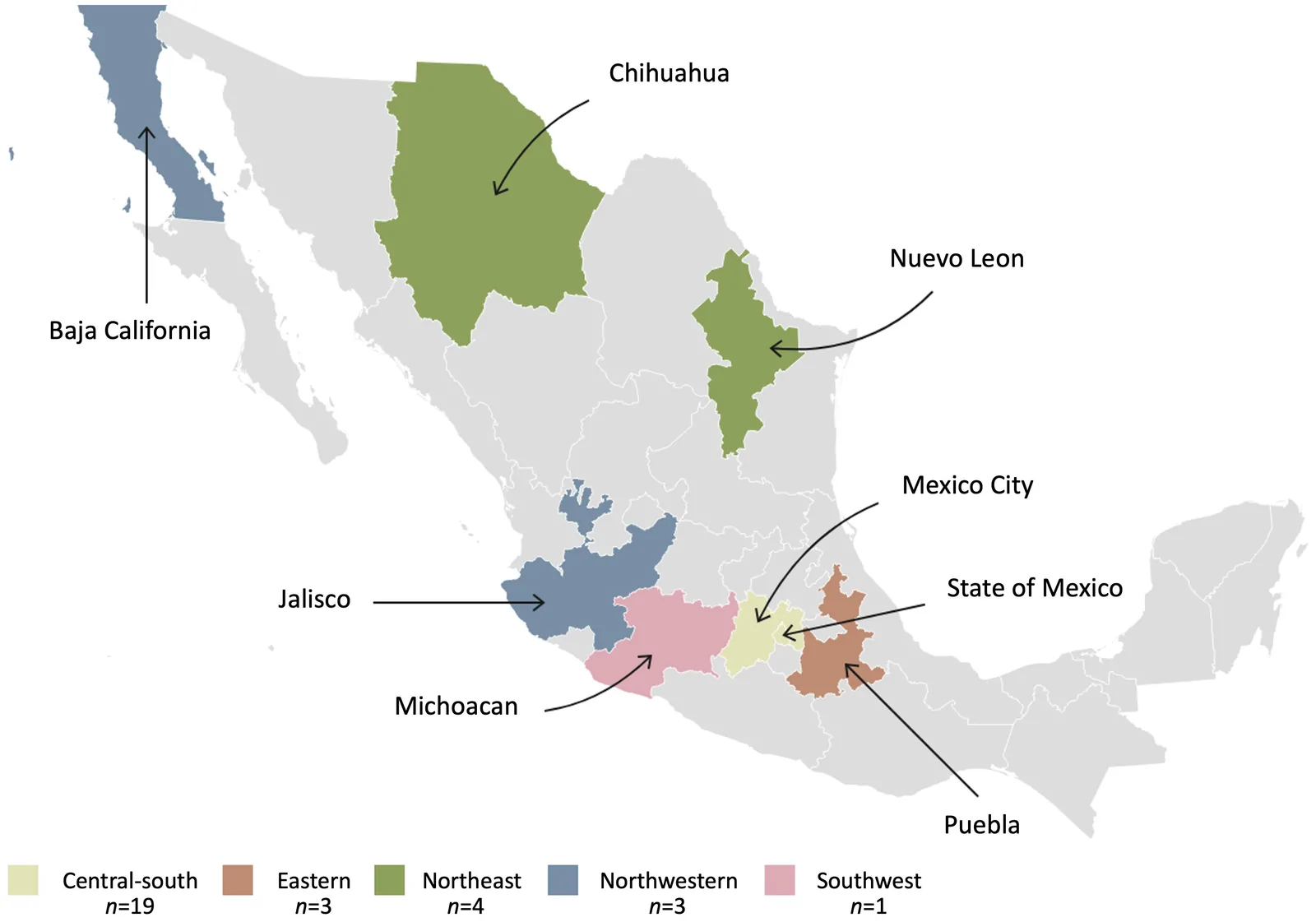
Geographic distribution of patients with GOF STAT1 disease. The states are grouped according to the geographic regions of Mexico, northwestern (blue), northeast (green), central-south (beige), eastern (terracotta) and southwest (pink).

**Table 1:** Demographic and genetic features in 30 Mexican patients with *STAT1* Gain-of-function pathogenic variants.

Kindred code	Patient code	Place of origin	Gender	Age at last visit	*STAT1* protein variant	Detection method	Protein domain
A	P1 (12)	Mexico City	M	85	E711Q	SS	TAD
A	P2 (12)	Mexico City	M	54	E711Q	SS	TAD
A	P3 (2, 12)	Mexico City	F	57	E711Q	SS	TAD
A	P4 (12)	Mexico City	F	59	E711Q	SS	TAD
A	P5 (12)	Mexico City	M	28	E711Q	SS	TAD
A	P6 (2, 12)	Mexico City	F	37	E711Q	SS	TAD
A	P7 (2, 12)	Mexico City	F	33	E711Q	SS	TAD
A	P8 (12)	Mexico City	F	30	E711Q	SS	TAD
A	P9	Mexico City	M	7	E711Q	SS	TAD
B	P10	Mexico City	F	23	R274W	GP	CCD
B	P11	State of Mexico	F	2	R274W	GP	CCD
C	P12	Mexico City	F	43	A267V	GP	CCD
C	P13	Puebla	F	22	A267V	GP	CCD
D	P14	Chihuahua	F	41	C324W	WES	DBD
D	P15	Chihuahua	M	11	C324W	GP	DBD
E	P16	Puebla	M	57	M202I	WES	CCD
E	P17	Mexico City	M	28	M202I	WES	CCD
F	P18	Michoacan	F	46	R274Q	SS	CCD
F	P19	Mexico City	M	24	R274Q	SS	CCD
G	P20	Mexico City	M	10	G384V	GP	DBD
H	P21 (10)	Mexico City	M	30	R321G	GP	DBD
I	P22	Jalisco	M	10	T288I	GP	CCD
J	P23 (11)	Puebla	M	5	N297del	GP	CCD
K	P24	Baja California	M	16	C324R	GP	DBD
L	P25 (13)	Nuevo León	F	7	E563Q	GP	L
M	P26	Mexico City	M	19	R274Q	GP	CCD
N	P27	Nuevo León	F	8	Y289C	GP	CCD
O	P28 (3)	Jalisco	F	37	T288A	SS	CCD
P	P29	Mexico City	M	36	Q243E	WES	CCD
Q	P30	Mexico City	F	2	A267V	GP	CCD

F, female; M, male; SS, Sanger sequencing; GP, gene panel; WES, whole-exome sequencing; TAD, transactivation domain; CCD, coiled-coil domain; DBD, DNA-binding domain; L, interdomain linker; Y, yes. Pathogenic variants are described using protein-level nomenclature. Previously reported patients are indicated by the corresponding reference number in superscript.

In the 30 included patients, the responsible pathogenic variant was identified using the following techniques: Sanger sequencing (*n* = 12), gene panels for IEI (*n* = 14), and whole-exome sequencing (*n* = 4) (**Table 1**). Fifteen different variants were identified across the 17 families; 14 had been previously reported (8), whereas the C324W variant identified in family D was novel. Functional studies demonstrated that the C324W *STAT1* variant conferred a GOF effect, expanding the mutational spectrum associated with this IEI (**Supplementary Figure 1**). Among the 15 pathogenic *STAT1* variants identified, 14 (93.3%) were missense variants, while only one (6.7%) corresponded to a non-frameshifting deletion (8). The 15 variants were distributed across multiple structural domains, predominantly within the coiled-coil domain (*n* = 9), followed by the DNA-binding domain (*n* = 4), with single variants identified in the Inter-domain Linker and transactivation domains (**Supplementary Figure 2**).

Of the 30 patients included in the cohort, 18 (60%) underwent assessment of serum immunoglobulin levels (IgG, IgA, IgM, and IgE) and T-cell subpopulations (CD3+, CD4+, and CD8+). Elevated IgG levels were observed in 56.2% of patients, while the remainder had values within the reference range. Increased IgA levels were present in 40% of patients. Most patients had normal IgM levels, with elevated and decreased concentrations observed in 26.7% and 6.6% of cases, respectively. Decreased CD3+ T-cell counts were observed in 14.3% of patients. CD4+ T-cell counts were reduced in 26.7% and elevated in 6.6%, while CD8+ T-cell counts were elevated in 6.6% of patients; the remaining values were within the reference range.

### Fungal infections

Fungal infections represented one of the most common clinical manifestations among the patients included in the study (6). CMC was observed in the 30 patients, with a median age at onset of 48 months (IQR: 7–180 months). Because adult patients often did not accurately recall CMC symptom onset, this variable was analyzed separately in the 22 patients younger than 12 years, showing an earlier median age at CMC onset of 12 months (IQR: 7–60 months). The distribution of age at CMC onset was as follows: <2 years, *n* = 13 (43.3%); 2–5 years, *n* = 5 (16.6%); and >5 years, *n* = 12 (40%). Most patients (90%, *n* = 27) presented involvement of two or more anatomical sites, whereas only 10% (*n* = 3) had disease restricted to a single site. CMC involved the oral mucosa in all patients, presenting as pseudomembranous candidiasis, angular cheilitis, stomatitis, glossitis, or candidal gingivitis. Other mucosal sites were affected, vulvovaginal candidiasis was documented in four female patients (26.6%), presenting at 20 years (P4), 5 years (P12), 3 years (P13), and 9 months of age (P28). Candidal balanitis was identified in two male patients (13.3%). However, the frequency may be underestimated, as patients in Mexico may be reluctant to disclose genital manifestations due to embarrassment when specifically questioned about them. Esophageal candidiasis was present in five patients (16.6%), all of whom had confirmed erosive esophagitis (P4, P16, P19, P21, P29). Cutaneous and adnexal involvement was frequent, nail involvement was one of the most common CMC manifestations, affecting 66.6% of patients (*n* = 20), all associated with onychodystrophy. Diaper-area involvement was documented in 6.6% of patients (*n* = 2), while other cutaneous areas were affected in 10% (*n* = 3).

Invasive fungal infections occurred in six patients (20%), with a median age at presentation of 16.5 years old (IQR: 8–27 years). These included disseminated histoplasmosis (P10, P28), pulmonary aspergillosis (P14), pulmonary infection due to *Cryptococcus neoformans, Aspergillus fumigatus, Aspergillus calidoustus*, and *Trichoderma longibrachiatum* (P20), cryptococcal meningitis caused by *Cryptococcus gattii* (P24) and, septic shock with isolation of *Salmonella* and *Candida* spp. (P27). Other noninvasive fungal manifestations included tinea capitis (13.3%, *n* = 4; P19, P21, P23, P25), tinea corporis (6.6%, *n* = 2; P16, P28), and seborrheic dermatitis associated with *Malassezia furfur* (P20) (**Table 2**). Overall, these findings confirm CMC as the hallmark fungal manifestation of STAT1 GOF disease while highlighting that patients from endemic regions may also develop life-threatening invasive mycoses. This broader fungal spectrum underscores the influence of regional epidemiology on the clinical phenotype of STAT1 GOF.

**Table 2:** Infectious manifestations in 30 Mexican patients with *STAT1* Gain-of-function pathogenic variants.

Patient code	Fungal infections	Bacterial infections	Viral infections
P1	Oral & nail CMC Glossitis		Shingles on the anterior chest & back
P2	Oral & nail CMC Cheilitis Stomatitis Glossitis	Urinary tract infections Scarlet fever	Lumbar herpes zoster (two events)
P3	Oral & nail CMC		Herpes zoster cervical, gluteal & lower extremity Oral herpes simplex
P4	Oral, nail, vulvovaginal & esophageal CMC Oral ulcers Glossitis	Sinusitis	Herpes simplex (groin & shoulder)
P5	Oral & nail CMC Oral ulcers Cheilitis Stomatitis		
P6	Oral & nail CMC		SARS-CoV-2 pneumonia
P7	Oral & nail CMC Oral ulcers Cheilitis Stomatitis	Endocarditis	Herpes Zoster Oral herpes simplex Influenza pneumonia
P8	Oral & nail CMC Oral ulcers Cheilitis	Urinary tract infections Cellulitis	Herpes zoster (left upper limb & axillary regions)
P9	Oral & nail CMC Oral ulcers		Herpetic whitlow
P10	Oral & nail CMC Hemophagocytic syndrome associated to disseminated histoplasmosis*		
P11	Oral & diaper area CMC		Influenza B, rhinoenterovirus & parainfluenza 3 pneumonia
P12	Oral, vulvovaginal & nail CMC	Urinary tract infections	SARS-CoV-2 pneumonia Influenza pneumonia
P13	CMC oral, vulvovaginal & nail	Urinary tract infections Pharyngotonsillitis	Oral herpes simplex SARS-CoV-2 pneumonia Influenza pneumonia
P14	Oral & nail CMC Oral ulcers Pulmonary aspergillosis		
P15	Oral & nail CMC Oral ulcers Stomatitis		
P16	Oropharyngeal & esophageal CMC Tinea corporis	Pneumonia with pleural effusion *(K. pneumoniae, E. coli, S. pneumoniae* & *P. aeruginosa)*	Oral herpes simplex Common warts
P17	Oral CMC	Mastoiditis	
P18	Oral & esophageal oral		
P19	Oral, nail & skin CMC Tinea capitis	Sinusitis Acute otitis media	
P20	Oropharyngeal & esophageal CMC Gingivitis Polymicrobial pulmonary fungal infection due to *Cryptococcus neoformans*, *Aspergillus fumigatus*, *Aspergillus calidoustus* & *Trichoderma longibrachiatum* Seborrheic dermatitis (*Malassezia furfur*)		
P21	CMC oral, nail Tinea capitis	Pneumonia (*Pseudomonas* spp.*, K. pneumoniae* & *Nocardia* spp.) Cutaneous abscesses	Chest & abdomen shingles
P22	Oral & glans penis CMC, Oral ulcers	Acute otitis media	Oral herpes simplex
P23	Oral CMC Tinea capitis	*S. aureus* sepsis	
P24	Oral & nail CMC *Cryptococcus gatti* meningitis		
P25	Oral & skin CMC Tinea capitis	Sinusitis Acute otitis media *S. aureus* pneumonia	Hemophagocytic syndrome associated to SARS-CoV-2 pneumonia*
P26	Oral, nail & diaper area CMC Cheilitis Stomatitis		Shingles Pneumonia caused by SARS-CoV-2
P27	Oral CMC *Candida* spp sepsis.	Septic shock with isolation of *Salmonella* & *Candida* spp. *	
P28	Oral, vulvovaginal, nail & skin CMC Oral ulcers Disseminated histoplasmosis* Tinea corporis	Pansinusitis	Chest shingles
P29	Oropharyngeal, glans penis & nail CMC Oral ulcers	*Helicobacter pylori* gastritis	Oral herpes simplex Hepatitis C virus infection
P30	Oral CMC Cheilitis	Pharyngotonsillitis	

P, patient; CMC, chronic mucocutaneous candidiasis; * This infection was the cause of death.

### Bacterial infections

Pyogenic bacterial infections are a prominent feature of STAT1 GOF disease, often characterized by recurrent and severe manifestations involving the respiratory tract, although other anatomical sites may also be affected (6, 8, 9). Seventeen of 30 patients (56.6%) had at least one bacterial infection. Severe invasive bacterial infections included endocarditis (P7), pneumonia with loculated pleural effusion caused by *Klebsiella pneumoniae, Escherichia coli, Streptococcus pneumoniae*, and *Pseudomonas aeruginosa* (P16), pneumonia caused by *Pseudomonas* spp., *Klebsiella pneumoniae*, and *Nocardia* spp. (P21), *Staphylococcus aureus* sepsis (P23), *Staphylococcus aureus* pneumonia confirmed in bronchoalveolar lavage fluid (P25), and septic shock with isolation of *Salmonella* and *Candida* spp. (P27). Five patients had bronchiectasis (P4, P14, P16, P17, P25). Other presumed bacterial infections without microbiological confirmation included recurrent otitis media (*n* = 3; 10%), sinusitis (*n* = 3; 10%), pharyngotonsillitis (*n* = 2; 6.6%), mastoiditis (*n* = 1; 3.3%), pansinusitis (*n* = 1; 3.3%), scarlet fever (*n* = 1; 3.3%), cellulitis (*n* = 1; 3.3%), and cutaneous abscesses (*n* = 1; 3.3%) (**Table 2**). Urinary tract infection by *E. coli* was documented in four patients, and *Helicobacter pylori* gastritis in one (P29). Overall, bacterial infections in STAT1 GOF disease were frequently recurrent and severe, leading to chronic pulmonary sequelae and, in some patients, concomitant fungal infections.

### BCG infection

In Mexico, BCG vaccination is mandatory at birth (18). Tweenty-four STAT1 GOF patients were vaccinated at the birth (83.3%) and one (P22) received BCG vaccine at 4 months of age. Five STAT1 GOF adult patients (17%; P1, P4, P12, P13, P18) were unaware of their vaccination status. Regarding vaccine strain, Danish 1331 was administered in 17 patients (68%) and Tokyo 172–1 in 8 (32%). BCG infection occurred in six of the 25 vaccinated patients (24%) (**Figures 3A–C**). Five patients (P20, P21, P23, P24, P27) developed BCG-itis, and one (P22) developed BCG-osis. The median age at onset of BCG-itis was 15 months (IQR: 4–18 months). One patient was categorized as culture-confirmed (P24), three as possible (P21, P23, P27), and one as probable (P20). One patient improved after right lymph node resection alone (23), three after lymph node resection combined with antimycobacterial drugs (P20, P22, and P24), and two with antimycobacterial therapy alone (P21 and P27). The antimycobacterial regimens and treatment durations were as follows: P20 received isoniazid, rifampicin, ethambutol, and clarithromycin for 20 months; P21 received rifampicin for 8 months; P24 received isoniazid and rifampicin for 9 months; and P27 received isoniazid and rifampicin for 3 months. At 18 months of age, one patient (P22) developed pulmonary BCG with positive PCR and confirmed by positive culture. This patient showed clinical improvement after an 8-month therapeutic regimen of rifampin, pyrazinamide, ethambutol, and isoniazid. Overall, these findings highlight BCG disease as an early and clinically important manifestation of STAT1 GOF.

**Figure 3:**
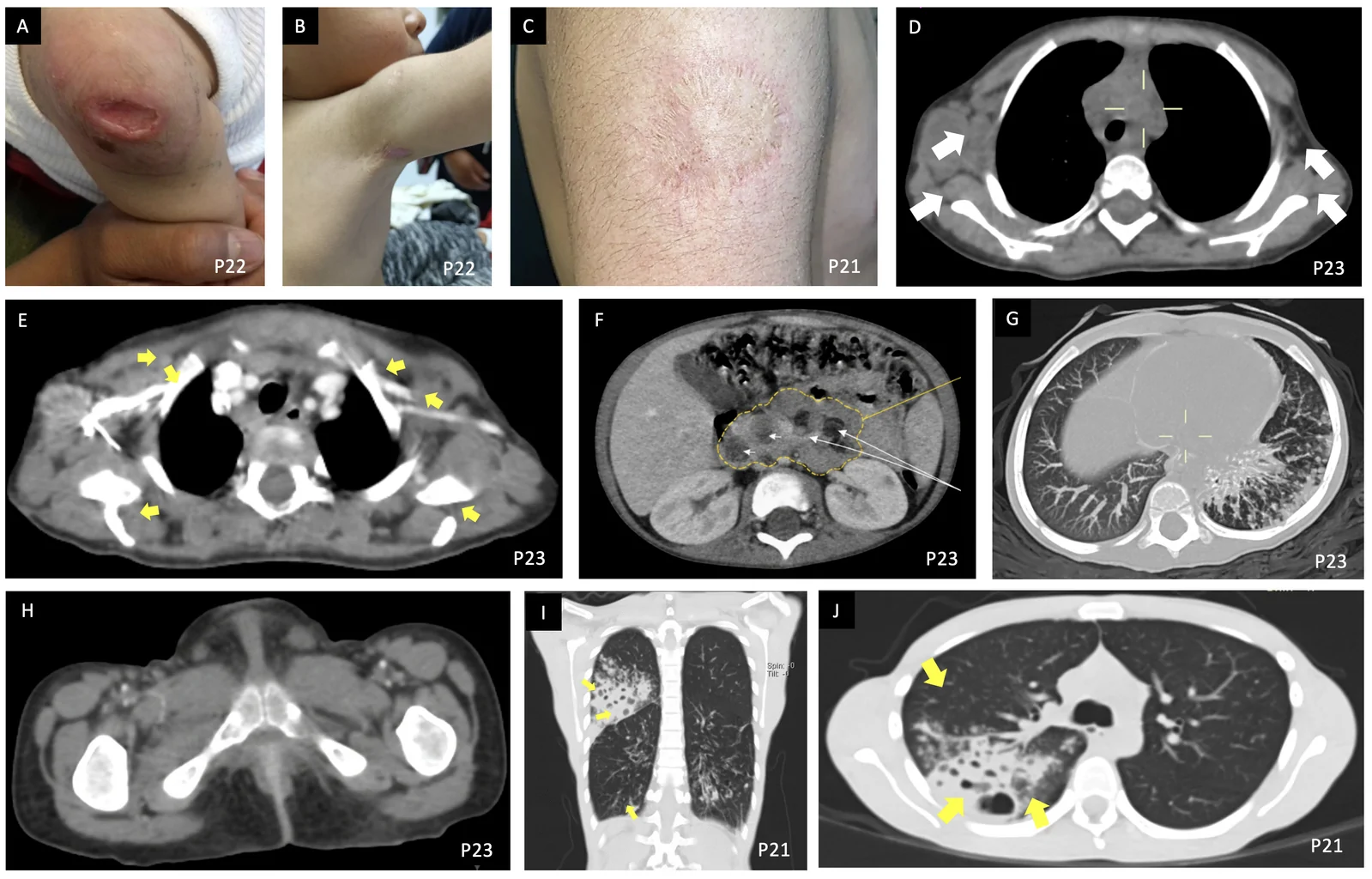
BCG-associated disease and tuberculosis in patients with GOF STAT1 disease. **(A)** Deep ulceration of the right shoulder following BCG vaccination. **(B, C)** Residual scars resulting from a localized BCG reaction in a 4-year-old **(B)** and a 30-year-old patient **(C)**. **(D–H)** Radiological Images in P23. **(D)** Axial contrast-enhanced chest computed tomography (CT), mediastinal window, demonstrating bilateral axillary lymphadenopathy, more pronounced on the right. **(E)** Axial contrast-enhanced neck CT showing multiple enlarged lymph nodes involving the bilateral lower cervical, supraclavicular, and superior mediastinal regions. **(F)** Axial contrast-enhanced abdominal CT obtained during the portal venous phase demonstrating a bulky retroperitoneal nodal conglomerate composed of multiple confluent lymph nodes, predominantly involving the right para-aortic and pericaval regions, with extension into the interaortocaval space. The nodal mass exhibits lobulated margins, heterogeneous attenuation, central hypoattenuating areas suggestive of necrosis, and mild peripheral contrast enhancement. **(G)** Axial chest CT, lung window, demonstrating segmental consolidation of the left lower lobe associated with multiple centrilobular micronodules, a tree-in-bud pattern, and peribronchiolar thickening, consistent with endobronchial dissemination. **(H)** Axial contrast-enhanced pelvic CT demonstrating enlarged lymph nodes in the left inguinal region. **(I, J)** Radiological Images in P23. **(I)** Coronal reconstruction of chest CT, lung window, demonstrating extensive cavitary consolidation of the right upper lobe with multiple thick-walled cavities, accompanied by bilateral centrilobular nodules and a tree-in-bud pattern, predominantly affecting both lower lobes. **(J)** Axial chest CT, lung window, showing cavitary consolidation of the right upper lobe with multiple thick-walled cavities, associated with adjacent centrilobular nodules and a tree-in-bud pattern.

### *Mycobacterium tuberculosis* infection

Toubiana et al. reported an international cohort of patients with STAT1 GOF disease, comprising 274 individuals, 62% of whom were from Europe, where the incidence of tuberculosis is low (6). Here, seven patients (23.3%; P1, P3, P7, P21, P23, P24 and P28) (**Figures 3D–J**) developed mycobacterial infections other than BCG; Six of the seven patients (P3, P7, P21, P23, P24, and P28) had documented BCG vaccination. Patients P21, P23, and P24 experienced both BCG infection and tuberculosis at distinct time points during their clinical course. According to the criteria proposed by Graham for classifying patients with tuberculosis, we obtained the following distribution: in P7, infection due to *Mycobacterium tuberculosis* was microbiologically confirmed by culture. In three additional cases (P21, P23, and P28), probable *M. tuberculosis* infection was identified using the GeneXpert MTB/RIF molecular assay. In three patients (P1, P3 and P24), the diagnosis was considered possible tuberculosis based on clinical criteria, radiological findings, and favorable response to anti-tuberculosis therapy. Pulmonary involvement was the most common presentation, observed in four patients (13.3%). In addition, P23 developed disseminated tuberculosis with axillary, hilar, mesenteric, retroperitoneal, and inguinal lymph node involvement, while other patients (P24 and P28) presented with both pulmonary and intestinal disease. Regarding antimycobacterial therapy, P1 received treatment in 1950; however, because this occurred many years ago, treatment details are unavailable. P3 received isoniazid, rifampicin, pyrazinamide, and ethambutol for 12 months. P2 received an intensive phase consisting of isoniazid, rifampicin, pyrazinamide, and ethambutol for 4 months, followed by a continuation phase with isoniazid and rifampicin for an additional 4 months. Similarly, P21 received an intensive phase with isoniazid, rifampicin, pyrazinamide, and ethambutol for 8 months, followed by a continuation phase with isoniazid and rifampicin for 2 months. P23 received isoniazid, rifampicin, pyrazinamide, and ethambutol for 12 months. P24 received isoniazid, rifampicin, pyrazinamide, and ethambutol for 18 months. P28 was treated with isoniazid, rifampicin, pyrazinamide, ethambutol, levofloxacin, and clarithromycin; however, treatment duration is unknown. All patients showed favorable clinical outcomes, with complete resolution of infection and no documented recurrent mycobacterial infections or relapses after treatment (**Table 3**). Non environmental mycobacterial has been documented in this cohort.

**Table 3:** Characteristics of tuberculosis (TB) infection in 30 patients with STAT1 Gain-of-function pathogenic variants.

Patient code	TB localization	Graham criteria TB classification	BCG infection background	TB regimen	TB regimen duration
P1	Pulmonary tuberculosis	Possible	No	Yes*	*
P3	Pulmonary tuberculosis	Possible	No	HZRE	12 mo
P7	Pulmonary tuberculosis	Confirmed	No	HZRE	4 mo intensive phase (HZRE) 2 mo continuation phase (HR)
P21	Pulmonary tuberculosis	Probable	Yes	HZRE	4 mo intensive phase (HZRE) 8 mo continuation phase (RH)
P23	Disseminated axillary, hilar, mesenteric, retroperitoneal, and inguinal lymphadenopathy	Probable	Yes	HZRE	12 intensive mo (HZRE)
P24	Pulmonary and intestinal dissemination	Possible	Yes	HZRE	18 mo
P28	Pulmonary and intestinal dissemination	Probable	No	HZRE+Lfx+ Clr	*

H, isoniazid; R, rifampicin; Z, pyrazinamide; E, ethambutol; Lfx, levofloxacin; Clr, clarithromycin; mo, months.

* Information on the medications administered was unavailable.

### Viral infections

Patients with STAT1 GOF disease are susceptible to recurrent and, occasionally, severe viral infections, particularly those caused by herpesviruses, reflecting impaired antiviral immune responses (6, 8). Viral infections were documented in 18 of 30 patients (60%), predominantly caused by the Herpesviridae family (*n* = 16). Herpes simplex infection was identified in 8 patients (26.6%), including oral (*n* = 6) and cutaneous (*n* = 2) disease. Herpes zoster occurred in 8 patients (26.6%), involving the cervical, axillary, thoracic, lumbar, abdominal, gluteal, upper, and lower extremity regions. One patient (P2) had two episodes affecting the same lumbar region, while another (P3) presented a single episode with multifocal involvement of the cervical, gluteal, and lower extremity regions. Respiratory viral infections included SARS-CoV-2 infection in 5 of 30 patients (16.6%); pneumonia was developed by two patients, one of whom was hospitalized. Four patients recovered, whereas a 5-year-old girl (P25) died from refractory hemophagocytic syndrome. Influenza pneumonia occurred in 3 patients (10%), and one additional patient developed a single episode of pneumonia associated with Influenza B virus, rhinovirus/enterovirus, and parainfluenza virus type 3. One patient developed hepatitis C virus infection, and another had human papillomavirus infection presenting as common warts on the fingertips (**Table 2**). Collectively, these findings highlight the broad clinical spectrum of viral infections in STAT1 GOF and emphasize their potential to trigger life-threatening complications such as hemophagocytic syndrome.

### Inflammatory and autoimmune manifestations

Immune dysregulation is a hallmark of STAT1 GOF disease, Inflammatory and autoimmune manifestations are common in these patients; however, some inflammatory manifestations may have an underlying autoimmune etiology (6, 8, 19–21). Autoinflammatory manifestations were observed in 15 patients (50%). Rosacea was the most frequent manifestation including phymatous (n=7 patients,23.3%), erythematotelangiectatic (n=6, 20%), papulopustular (n=4,13.3%), and ocular rosacea (n=8, 26.6%). Documented childhood-onset rosacea was observed only in P23, one of the patients included in the present cohort. Although age at onset was unavailable for the remaining six subjects, the median age at documentation among surviving patients was 33.5 years (IQR: 23.5–55.5) (**Figure 4**). Other autoinflammatory manifestations included intestinal vasculitis with two perforation episodes in P2, pancreatitis in P1, and generalized exfoliative dermatitis in P4 (**Table 4**). Two patients developed infection-triggered macrophage activation syndrome: P10 secondary to histoplasmosis and P25 secondary to SARS-CoV-2 infection. Autoimmune manifestations were identified in 12 patients (40%), five were females (42%); seven patients (58%) had two or more autoimmune manifestations, whereas the remaining patients had only one. Age at onset ranged from 1 to 37 years, with a median age at diagnosis of 8 years (IQR: 5–26.5). Additional autoimmune manifestations included hypothyroidism (P4, P6, P8, P9, P15, P20, P22, P23, P25), systemic lupus erythematosus (P8 and P12), type 1 diabetes mellitus (P15), latent autoimmune diabetes in adults (P4), autoimmune atrophic gastritis (P2), pernicious anemia (P2), autoimmune enteropathy (P4), autoimmune hepatitis (P6), alopecia areata (P20), vitiligo (P28), discoid lupus erythematosus (P4), and antiphospholipid syndrome (P28) (**Table 4**). The four pediatric patients with hypothyroidism (P9, P22, P23, P25) presented with short stature (< –2 SD). Analysis of anti-TPO and anti-Tg antibodies revealed a heterogeneous pattern within the study group (**Table 5****).** Overall, these findings underscore the broad spectrum of immune dysregulation in STAT1 GOF and the need for long-term multidisciplinary follow-up, particularly given the potential fatality of complications such as macrophage activation syndrome.

**Figure 4:**
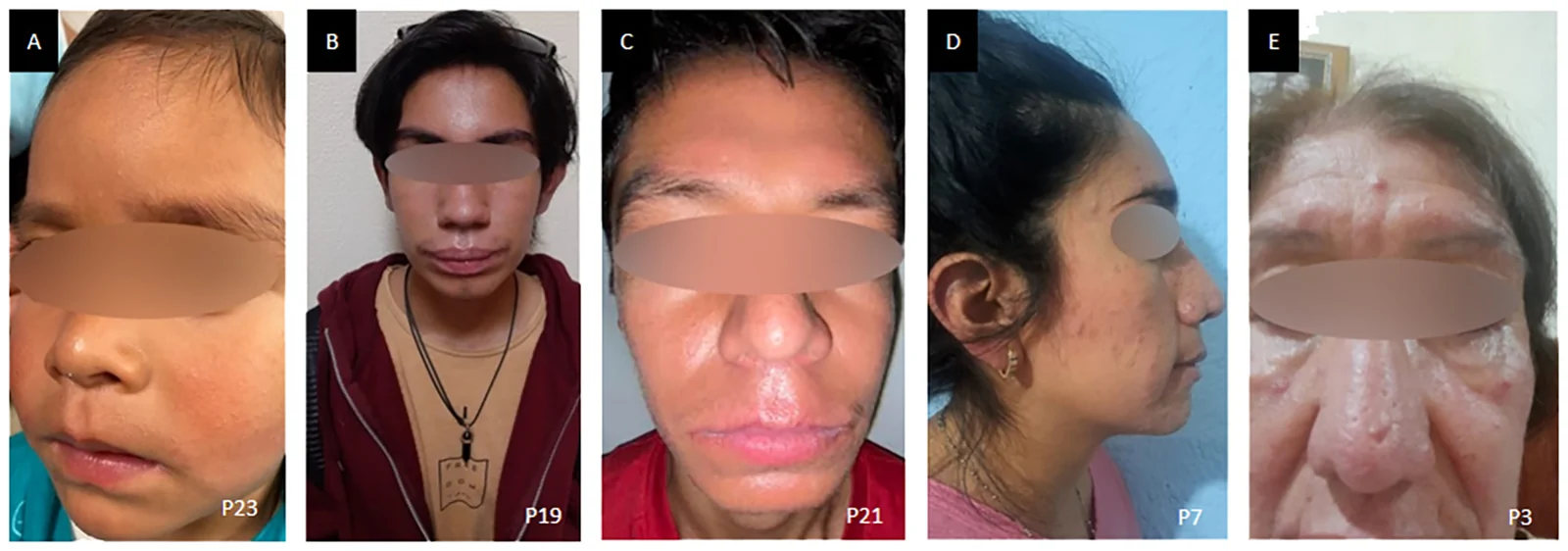
Dermatological manifestations in patients with GOF STAT1 disease. **(A–E)** Facial rosacea in patients of different ages.

**Table 4:** Autoinflammatory, autoimmune, and malignant manifestations in 30 Mexican patients with *STAT1* Gain-of-function pathogenic variants.

Patient code	Autoinflammatory manifestations	Autoimmune manifestations	Cancer	Miscellany
P1	Phymatous rosacea Pancreatitis		Prostate cancer Oral squamous cell carcinoma	
P2	Phymatous & ocular rosacea Recurrent intestinal vasculitis (perforation)	Pernicious anemia Atrophic gastritis		
P3	Phymatous & ocular rosacea			
P4	Phymatous & ocular rosacea Generalized desquamative dermatitis	Latent autoimmune diabetes in adults Autoimmune enteropathy Discoid lupus erythematosus Hypothyroidism	Esophageal squamous cell carcinoma	Bronchiectasis Erosive esophagitis
P6	Phymatous & papulopustular rosacea	Autoimmune hepatitis Hypothyroidism		
P7	Papulopustular rosacea			
P8	Phymatous, ocular & papulopustular rosacea	Systemic lupus erythematosus Hypothyroidism		
P9		Hypothyroidism		Short stature
P10	Hemophagocytic syndrome			
P12		Systemic lupus erythematosus		
P14				Bronchiectasis
P15		Type 1 diabetes mellitus Hypothyroidism		
P16				Bronchiectasis Erosive esophagitis
P17	Erythematotelangiectatic rosacea			Bronchiectasis
P18			Esophageal squamous cell carcinoma	
P19				Erosive esophagitis
P20	Erythematotelangiectatic rosacea	Alopecia areata Hypothyroidism		
P21	Ocular & erythematotelangiectatic rosacea		Papillary thyroid carcinoma	Erosive esophagitis
P22		Hypothyroidism		Short stature
P23	Ocular & erythematotelangiectatic rosacea	Hypothyroidism		Short stature
P25	Hemophagocytic syndrome	Hypothyroidism		Bronchiectasis Short stature
P26	Ocular & erythematotelangiectatic rosacea			
P28		Antiphospholipid syndrome Vitiligo		
P29	Ocular, phymatous, erythematotelangiectatic & papulopustular rosacea			Erosive esophagitis
P30				

**Table 5:** Thyroid disease in 30 Mexican patients with *STAT1* Gain-of-function pathogenic variants.

Patient code	Disease	Age of onset (YO)	Thyroid Autoantibodies		Treatment	Current age (YO)
P4	Hypothyroidism	37	Anti TPO (–)	Anti Tg (–)	Levothyroxine	Deceased
P6	Hypothyroidism	31	*	*	Levothyroxine	37
P8	Hypothyroidism	22	Anti TPO (–)	Anti Tg (–)	Levothyroxine	30
P9	Hypothyroidism	6	Anti TPO (+)	Anti Tg (–)	Levothyroxine	7
P15	Hypothyroidism	9	Anti TPO (+)	Anti Tg (+)	Levothyroxine	11
P20	Hypothyroidism	7	Anti TPO (–)	Anti Tg (–)	Levothyroxine	10
P21	Papillary thyroid carcinoma	30	Anti TPO (–)	Anti Tg (–)	Levothyroxine	31
P22	Hypothyroidism	8	Anti TPO (–)	Anti Tg (–)	Levothyroxine	10
P23	Hypothyroidism	4	Anti TPO (–)	Anti Tg (–)	Levothyroxine	6
P25	Hypothyroidism	1	*	*	Levothyroxine	Deceased

YO, years old (–); negative result; (+) positive result; *not mesured; Anti-TG, Anti-thyroglobulin; Anti-TPO, anti-peroxidase.

### Malignancies

Malignancies have been increasingly recognized as a complication of STAT1 GOF disease, particularly squamous cell carcinomas, likely related to chronic mucosal inflammation, persistent viral infections, and immune dysregulation (8). Five malignancies were identified in four patients (P1, P4, P18, and P21). Patient P1 developed prostate cancer at 60 years of age, which responded to treatment, and later developed oral squamous cell carcinoma at 85 years of age; he died one month after diagnosis. Patients P4 and P18 developed esophageal squamous cell carcinoma at 59 and 46 years of age, respectively, both refractory to treatment. Patients with oral squamous cell carcinoma and esophageal squamous cell carcinoma had a history of treatment-refractory CMC. At 30 years of age, patient P21 was diagnosed with papillary thyroid carcinoma and underwent a total thyroidectomy combined with lymph node dissection. Prior to surgery, thyroid function tests and thyroid autoantibodies (anti-TPO/anti-Tg) were within normal limits and negative, respectively; today, the patient remains on levothyroxine therapy with a good clinical response (**Table 5**). Overall, these findings highlight the increased risk of malignancy in STAT1 GOF, particularly in patients with persistent mucocutaneous inflammation and chronic infections, supporting the importance of long-term surveillance and early cancer detection strategies.

### Mortality

Despite advances in the recognition and treatment of STAT1 GOF disease, mortality remains an important outcome, mainly driven by severe infections, hyperinflammatory complications, and malignancies. Assessment of causes of death provides insight into the major determinants of disease severity in affected patients (8). Seven of the 30 patients died, corresponding to an overall mortality rate of 23.3%. The median age at death was 37 years (IQR: 8–59). Among the deceased patients, malignant neoplasms were responsible for three deaths: oral squamous cell carcinoma in P1 (aged 85), esophageal squamous cell carcinoma in P4 (aged 59), and esophageal cancer in P18 (aged 46). Infectious complications accounted for the remaining four cases, including SARS-CoV-2 associated to hemophagocytic syndrome in P25 (aged 7), septic shock with isolation of *Salmonella* and *Candida* spp. in P27 (aged 8), disseminated histoplasmosis associated to hemophagocytic syndrome in P10 (aged 23) and, cerebral histoplasmosis in P28 (aged 37). None of the patients underwent hematopoietic stem cell transplantation (HSCT). Notably, mortality in this cohort was associated with both malignant neoplasms and severe infectious complications, including invasive fungal disease and infection-triggered hemophagocytic syndrome, highlighting the broad clinical spectrum and life-threatening complications of STAT1 GOF.

## Discussion

We describe the first Mexican cohort of patients with STAT1 GOF disease, representing a single-country study that includes children, adults, and elderly individuals. Among this cohort of 30 patients harboring 15 different pathogenic variants, only one had previously been reported as a variant of uncertain significance (VUS). However, functional studies demonstrated that the C324W STAT1 variant confers a GOF effect. Thirty-six percent of patients were *de novo* cases, whereas the remaining individuals belonged to familial cases, highlighting the importance of extending genetic evaluation to relatives; notably, family A included nine affected members. Regarding fungal infections, the penetrance of CMC was consistent with previously reported cohorts; however, our study revealed a higher frequency of invasive fungal infections compared with other series (20% vs. 10%) (6, 9). This difference may be explained by the environmental distribution of endemic fungi, which is shaped by regional climatic conditions, soil composition, and specific ecological niches (22, 23). Invasive fungal infections reported in STAT1 GOF patients include *Histoplasma* spp., *Cryptococcus* spp., *Coccidioides* spp., *Aspergillus* spp., *Pneumocystis jirovecii*, and *Penicillium marneffei* (5, 24). In this study, three patients developed invasive fungal infections, with two fatal cases of histoplasmosis and one case of cryptococcosis. These findings highlight that the infectious phenotype of STAT1 GOF is shaped not only by the underlying immune defect but also by regional epidemiological factors, including exposure to geographically restricted fungal pathogens.

One of the most relevant findings of our cohort was the high frequency of mycobacterial infections. Mexico is a country with a high tuberculosis burden, with an estimated incidence of 16 cases per 100,000 population, and universal neonatal BCG vaccination is part of the national immunization program (25). Among vaccinated patients, 24% developed BCG disease, whereas 23% developed tuberculosis. These frequencies are substantially higher than those reported in international cohorts (6, 8, 9) and contrast with a Norwegian cohort of 18 patients with STAT1 GOF, in which no mycobacterial infections were observed (10). Norway is among the countries with the lowest tuberculosis incidence, and routine BCG vaccination was discontinued in 2009 (26, 27). In contrast, a cohort from Morocco, a country where tuberculosis (TB) remains a major public health challenge, reported an average annual TB incidence of 113 cases per 100,000 population (28). Bouchra Baghad et al. reported mycobacterial infections in five individuals (41.67%), which included recurrent pulmonary tuberculosis, pleuropulmonary tuberculosis, a tuberculous hepatic abscess, tuberculous lymphadenitis, and localized bacillus Calmette–Guérin (BCG)–related infection in a pediatric patient (29). Also, Zimmerman et al. reported mycobacterial infections in 20% of 35 patients with STAT1 GOF from the United States, most of whom had infections caused by the *Mycobacterium avium* complex, whereas only one patient developed *Mycobacterium tuberculosis* infection (30). In our cohort, the spectrum of mycobacterial disease included isolated BCG disease, isolated tuberculosis, and patients who developed tuberculosis years after a previous episode of BCG disease. Clinical presentations ranged from localized to disseminated infection, and all patients responded favorably to antimycobacterial therapy without relapse or recurrent disease. *Mycobacterium tuberculosis* infection occurred across a wide age range, from infancy to adulthood. It is important to note that, according to the Graham criteria, the seven patients with tuberculosis were classified as confirmed (n=1), possible (n=3), and probable (n=3) cases. Notably, BCG disease was among the earliest clinical manifestations of STAT1 GOF and, in some patients, preceded the onset of CMC. No nontuberculous mycobacterial infections were identified; however, this finding should be interpreted with caution, as limited access to specialized diagnostic methods may have reduced their detection. In patients presenting with mycobacterial infection and a novel STAT1 variant, functional characterization is essential to determine whether the variant results in a gain- or loss-of-function effect, as these distinct mechanisms are associated with different clinical phenotypes. The mechanism of mycobacterial disease remains unknown.

Among pyogenic bacterial infections, invasive disease caused by *Klebsiella pneumoniae* and *Escherichia coli* has not been previously emphasized in STAT1 GOF, further expanding the spectrum of bacterial pathogens associated with this disorder. In this cohort, polymicrobial bacterial infections were observed during individual infectious episodes, highlighting the importance of comprehensive microbiological investigation. Therefore, identification of a single pathogen should not preclude further investigation for additional bacterial or fungal microorganisms.

Okada et al. proposed that STAT1 GOF may represent not only an immunodeficiency associated with recurrent infections but also an interferonopathy in which dysregulated interferon signaling may contribute to chronic inflammation (4). In our cohort, rosacea was the most common autoinflammatory manifestation, occurring from childhood through late adulthood. In patients with STAT1 GOF, cutaneous and ocular rosacea are frequently misdiagnosed as acne, pustular eruptions, furunculosis, folliculitis, or chronic conjunctivitis (31). Because rosacea may represent the earliest visible manifestation of the disease, STAT1 GOF should be considered in patients presenting with cutaneous or ocular rosacea in association with CMC. Macrophage activation syndrome (MAS) is a life-threatening hyperinflammatory disorder that may be triggered by infections in patients with IEI (32, 33). In our cohort, two patients developed fatal MAS, one triggered by SARS-CoV-2 infection and the other by disseminated histoplasmosis. These observations underscore the importance of close monitoring for hemophagocytic lymphohistiocytosis (HLH) diagnostic criteria during moderate or severe infections to enable early recognition and timely intervention before irreversible organ damage occurs. In contrast, no aneurysms were identified in our cohort. However, this finding should be interpreted with caution because none of our patients underwent systematic screening for asymptomatic vascular lesions. Therefore, vascular surveillance should be considered, particularly in patients with longstanding disease or systemic inflammatory manifestations.

In a systematic review by Zhang et al., 61% of patients with STAT1 GOF developed autoimmune disease (9). In our cohort, autoimmune manifestations were identified in 40% of patients, without a clear sex predominance, and most autoimmune disorders occurred during adulthood. Hypothyroidism was the most common autoimmune manifestation, affecting 30% of all patients and occurring in both children and adults. Not all patients with hypothyroidism had positive antithyroid autoantibodies. Importantly, thyroid function had not been assessed in all patients at the time of this study. We therefore recommend periodic monitoring of thyroid function throughout life, as hypothyroidism may develop from early childhood to late adulthood. Malignancies were identified in 13.3% of patients, a higher proportion than the 5.9% reported in the systematic review by Zhang et al. (9). Oral and esophageal squamous cell carcinomas were the predominant malignancies, and all three affected patients had a history of treatment-refractory chronic mucocutaneous candidiasis at the site where the carcinoma subsequently developed. These findings suggest that patients with persistent CMC are at increased risk of squamous cell carcinoma and support the implementation of surveillance strategies, including upper gastrointestinal endoscopy when clinically indicated, to facilitate early detection. STAT1 GOF should be considered in patients with BCG disease and tuberculosis, particularly in high-burden countries as Mexico. Several considerations should be taken into account when interpreting these findings, including the retrospective and multicenter design, the relatively small sample size, and the availability of clinical and immunological data. Recall bias regarding age at disease onset and potential referral bias may also have influenced some findings. In addition, microbiological confirmation was not available for all mycobacterial infections. These considerations should be kept in mind when interpreting the reported frequencies of infections and other clinical complications. Our findings suggest that the clinical phenotype of STAT1 GOF is determined not only by the underlying genetic variant but also by environmental and epidemiological factors.These considerations are essential for understanding the phenotypic variability of STAT1 GOF across different geographic regions.

## Data Availability

The original contributions presented in the study are included in the article/**Supplementary Material**. Further inquiries can be directed to the corresponding author.

## Supplementary material

Supplementary Figure 1 **(A)** *In vitro* functional studies to demonstrate a GOF effect in *STAT1*. GAS reporter activity of WT and variant STAT1 in U3C cells after 16 h of stimulation with the indicated concentrations of IFN-γ. Empty vector (EV), Y701C, and R274Q served as mock, loss-of-function, and gain-of-function controls, respectively. Each dot represents one well; bars show the mean ± SEM across three independent experiments, each performed in triplicate (n = 3 independent experiments). Statistical significance was assessed using the Holm–Bonferroni method. Comparisons with WT with *p* < 0.05 are indicated by an asterisk (*).

Supplementary Figure 2 **(B)** Distribution of 15 *STAT1* Gain-of-function mutations on the gene. The variants are located throughout the functional domains of the *STAT1* protein: The N-terminal domain (NTD), the coiled-coil domain (CCD), the DNA-binding domain (DBD), the linker domain (L), the SH2 domain (SH2D), the tail segment domain (TSD) and the transactivation domain (TAD). *Novel pathogenic variant.
